# Epigenetic differences in monozygotic twins discordant for major depressive disorder

**DOI:** 10.1038/tp.2016.101

**Published:** 2016-06-14

**Authors:** K Malki, E Koritskaya, F Harris, K Bryson, M Herbster, M G Tosto

**Affiliations:** 1 King's College London, MRC Social, Genetic and Developmental Psychiatry Centre at the Institute of Psychiatry, Psychology and Neuroscience, London, UK; 2 Department of Computer Science, University College London, London, UK; 3 Laboratory for Cognitive Investigations and Behavioural Genetics Tomsk State University, Tomsk, Russia

## Abstract

Although monozygotic (MZ) twins share the majority of their genetic makeup, they can be phenotypically discordant on several traits and diseases. DNA methylation is an epigenetic mechanism that can be influenced by genetic, environmental and stochastic events and may have an important impact on individual variability. In this study we explored epigenetic differences in peripheral blood samples in three MZ twin studies on major depressive disorder (MDD). Epigenetic data for twin pairs were collected as part of a previous study using 8.1-K-CpG microarrays tagging DNA modification in white blood cells from MZ twins discordant for MDD. Data originated from three geographical regions: UK, Australia and the Netherlands. Ninety-seven MZ pairs (194 individuals) discordant for MDD were included. Different methods to address non independently-and-identically distributed (non-i.i.d.) data were evaluated. Machine-learning methods with feature selection centered on support vector machine and random forest were used to build a classifier to predict cases and controls based on epivariations. The most informative variants were mapped to genes and carried forward for network analysis. A mixture approach using principal component analysis (PCA) and Bayes methods allowed to combine the three studies and to leverage the increased predictive power provided by the larger sample. A machine-learning algorithm with feature reduction classified affected from non-affected twins above chance levels in an independent training-testing design. Network analysis revealed gene networks centered on the *PPAR*−*γ* (*NR1C3*) and *C-MYC* gene hubs interacting through the *AP-1* (*c-Jun*) transcription factor. *PPAR*−*γ* (*NR1C3*) is a drug target for pioglitazone, which has been shown to reduce depression symptoms in patients with MDD. Using a data-driven approach we were able to overcome challenges of non-i.i.d. data when combining epigenetic studies from MZ twins discordant for MDD. Individually, the studies yielded negative results but when combined classification of the disease state from blood epigenome alone was possible. Network analysis revealed genes and gene networks that support the inflammation hypothesis of MDD.

## Introduction

Major depressive disorder (MDD) is a pervasive psychiatric disorder characterized by a number of clinical symptoms including: persistent low mood, anhedonia, insomnia, low energy, feelings of guilt and ideation of death or suicide.^1, 2^ MDD is also associated with a range of social impairments, including educational and occupational problems and with an increased risk of developing systemic disease, such as cardiovascular disease and Type 2 diabetes.^3^ Epidemiological studies have shown links between MDD and increased levels of mortality due to either suicide or resulting diseases.^4, 5^

Behavioral genetic research into the etiology of depression reports heritability estimates between 31 and 42% (refs 6, 7, 8) but uncovering common sequence variants associated with the pathology has been challenging. To date, there are no common genetic variants of sufficiently high penetrance to account for the pathology that have clinical significance, although variants associated with the disease at the genome-wide level have been recently announced.^9^ Environmental factors, such as early- and late-life stressors, are also thought to increase the risk of developing depression; however, the interaction between genetic and environmental factors remains poorly understood.^10^

Studying depression from the epigenetic perspective sheds light on its etiology by uncovering how environmental factors modulate gene expression.^11^ Epigenetics is the study of cellular modification and differentiation that is independent of alterations in the DNA sequence. This can be defined simply as the variation in phenotypic expression produced by the up- and downregulation of genes using DNA methylation and histone modification, rather than changes in the DNA sequence. It has been suggested that through these processes environmental factors can cause lasting changes in gene expression.^12^ For example, early-life stress leads to epigenetic changes in neural tissue involved in the stress response in both animal and human studies.^13, 14^

Quantitative genetics research typically compares phenotypic concordance rates between monozygotic (MZ) and dizygotic twins. However, discordant MZ twins (who are assumed to share 100% of their genome) can also be used to identify epigenetic factors, such as differing levels of DNA methylation at specific loci that may contribute to explain part of their phenotypic discordance.^15^

The genome (the DNA sequence) is consistent throughout all cell types in the human body: hence, buccal epithelial cells are largely used in genetic research because of their convenience. However, epigenetic changes are more tissue-specific. In the field of psychiatry, this presents a problem as, although neural tissues of interest can be obtained post-mortem, in live patients, only cells in peripheral tissues can generally be obtained. A recent study by Oh *et al.*^16^ successfully replicated multiple epigenetic features of depression between brain and non-brain tissues, suggesting that peripheral blood cells are adequate for investigating the epigenetics of depression.^16^ Studies using peripheral blood cells may also have an advantage in that they may show fewer changes associated with disease-related external factors, including exposures to stress and pharmacological treatments.^17^ On the other hand, it is likely that disease-relevant epigenetic variations may be considerably weaker in peripheral blood cells than in the neural tissue of interest or even be undetectable.

The study by Oh *et al.*^16^ analyzed epigenetic variations across different tissues in three data sets of discordant MZ twin pairs from three centers in different countries in order to identify epigenetic markers associated with depression.^16^ However, when they combined the data sets collected from blood, they were not able to discriminate affected siblings. This study used the same three previously published peripheral blood data sets from MZ twins discordant for MDD, aiming to address some of the statistical challenges of this data set to produce a global classification model. This required several data consolidation issues to be addressed. First, batch effects may lead to the data not being independently and identically distributed (non-i.i.d.); this refers to error introduced by systemic differences in the ways samples are taken and analyzed between different laboratories. The second refers to population stratification and noise variance. This study aims to explore methods to combine these data sets, adjusting for sources of error and then use the increased power of the combined data to classify affected and non-affected individuals according to epigenetic markers from peripheral blood samples of MZ twins discordant for MDD.

## Materials and methods

### Design

Merging raw data often violates independent-and-identically distributed (i.i.d.) assumptions and thus leads to biased model parameter estimations, higher generalization error and lower cross-validation model performance. The study first used an atheoretical, data-driven approach to evaluated methods for batch effect removal and non-i.i.d. data consolidation. In the second part of the study, we used two different machine-learning approaches (linear support vector machines (SVMs) and random forest (RF)) in order to build a model that could classify affected siblings from non-affected ones using the merged data set from the three, independent, cross-country, epigenetic studies described below. Levels of methylation were used as features and affected and non-affected status within each discordant twin pair as outcome labels. Training and testing of the algorithms was performed on independent samples (80:20% split). Feature selection methods for each algorithm were chosen based on cross-validation. Finally, several different thresholds for the number of selected features were evaluated to extract epigenetics markers likely to contribute to variation in the pathology. These features were then carried forward for gene-network analysis.

### Sample and variable description

The data used for this study have been obtained from a recently published study by Oh *et al.*, and are available from the Gene Expression Omnibus (accession number GSE37579). Briefly, methylation profiles were obtained using Illumina 8.1-K CpG island microarrays from white blood cells sampled from MZ twins discordant for MDD. The samples originated from three different regions: the United Kingdom (28 samples), Australia (80 samples) and the Netherlands (86 samples). Levels of methylation were measured for either coding or noncoding DNA regions common across the samples. Following quality control procedures, a total of 8448 features with log-normalized levels of methylation for each sample were obtained that were consistent across the three studies. Depending on the source of information, depressive symptoms were evaluated by structured clinical interview. A diagnosis of MDD was determined by the results of these questionnaires according to the Diagnostic and Statistical Manual of Mental Disorders, 4th Edition criteria. Australian participants were drawn from the Australian Twin Registry. Forty pairs were selected for this study, including 31 female pairs. The average age of the twins was 41.2 years, with a s.d. of 11.5 years. Dutch participants were drawn from the Netherlands Twin Register. Forty-three twin pairs were selected for this study, with an average age of 38.4 years and a s.d. of 12.7 years. UK participants were drawn from the St Thomas's Hospital Twin Registry, the Maudsley Hospital (London) Twin Register and from ongoing studies of volunteer twins. Fourteen pairs including two males were selected for this study. The average age of participants was 53.7 years, with a range from 21 to 65 years. This data set has been chosen for several key reasons. First, it is based on MZ discordant twins, which are considered matched for: genetics, age, sex, cohort effects, maternal influences and common environments. By design, MZ studies have more power to detect disease-related epigenetic differences than studies on unrelated individuals. Second, the same Illumina array has been used across the three studies avoiding potential confounds resulting from matching markers on different arrays and artefacts injected by imputation methods. Lastly, Oh *et al.* reported that, using all the twin samples (UK, Australian and Dutch), they were unable to discriminate affected individuals from control subjects from blood. Given their promising results from other tissue, there was good reason to believe that important information could still be gained from this published data set. Further information on the samples, ethical statements and approval data collection and data pre-processing is described elsewhere.^16^

### Statistical analysis

Empirical non-i.i.d. data consolidation methods, including principal component analysis (PCA) and non-parametric Bayes methods (ComBat), have been evaluated in isolation and in combination to batch effect removal, population stratification and other noise variance. Machine-learning methods and feature selection using linear SVMs and RF were used to build a classifier to predict cases and controls for MDD from epigenetic markers and extract features with higher probability of explaining variations in pathology.

### Data normalization and evaluation metric

Two main approaches of batch effect removal were evaluated in isolation and in combination. The PCA method relies on the idea that the direction with higher variance might relate to noise or population stratification rather than disease. The non-parametric ComBat approach is an empirical Bayes method that aims to adjust for unknown, unmodeled or latent sources of noise and systematic bias. MZ twin studies discordant on any phenotype are intrinsically balanced by nature: in each twin pair, we have an affected and non-affected twin. This balance was preserved in the training and test data sets during the resampling procedure via randomly sampling from twin pairs rather than from the whole data set. Although receiver operating characteristic (ROC) returns generally higher values, in this study accuracy was preferred as a more representative, conservative and honest measure of model performance.

### Data consolidation approaches

PCA, ComBat and mixture approaches were evaluated to address issues of non-i.i.d. data and control for potential confounding effects. The PCA method relies on the notion that eigenvectors with higher variance relate to subgroup phenotypes as opposed to disease groups. This approach removes unwanted variance by subtracting a matrix achieved via eigenvector decomposition. Removal of unwanted variance could relate to the removal of batch effect as described by the paper by Nielsen *et al.*,^18^ and to control for population stratification as described by Price *et al.*^19^ This study considered the approaches above, and unwanted variance was removed from each data set before merging data into one larger set as follows.

For each region *i*, data were adjusted as follows: 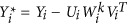, which represents the eigenvector decomposition of the data matrix. Matrices *U* and *V* contain the top *k* eigenvectors corresponding to the top *k* eigenvalues given in *W*. *Y* represents gene expression and *W*_*i*_^*k*^ is a diagonal matrix corresponding to the top *k* eigenvalues. For the purpose of evaluation we subtracted the matrix related to the most informative principal components ranked by eigenvalue. Therefore, *PCA.1* subtracts the matrix related to the first principal component where *PCA.2, PCA.3, PCA.4* and *PCA.5* sequentially subtracted the matrix related to principle components 2–5.

The second approach was based on the non-parametric ComBat method implemented in the Surrogate Variable Analysis 'sva' R-package available from Bioconductor.^20^ This is an empirical Bayes method aimed to adjust for unknown, unmodeled or latent sources of noise. ComBat adjusts for systematic batch bias common across genes, assuming that batch effect factors often affect many genes in similar ways, similar to increased expression or higher variability. The other benefit of adjusting for systematic bias with ComBat is that it robustly adjusts batch bias for even small batch sizes.^21^ ComBat is a three-step empirical Bayes method: (1) standardization of the data is achieved using the formula:


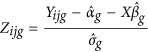


where 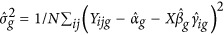 and 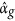, 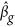, 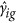 are estimations of parameters *α_g_*, *β_g_*, *γ*_*ig*_ in a model


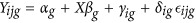


where *N* is the total number of samples, *m* is number of batches, *n*_*i*_ is number of samples within a batch *i* for *i*=1,…,*m*, for genes *g*=1,…,*G*. *Y*_*ijg*_ represents the expression value for gene *g* for sample *j* from batch *i*, *α*_*g*_ is the overall gene expression, *X* is a design matrix for sample conditions and *β*_*g*_ is the vector of regression coefficients corresponding to *X*. The error terms, 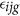, can be assumed to follow a Normal distribution with expected value of zero and variance 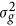. The *γ*_*ig*_ and *δ*_*ig*_ represent the additive and multiplicative batch effects of batch *i* for gene *g*, respectively.

(2) Batch effect parameters are estimate batch using empirical priors. Assuming that standardized data *Z*_*ijg*_~*N*(*γ*_*ig*_,*σ*_*ig*_) are normally distributed, the non-parametric estimates are:


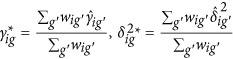


where 
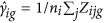
, 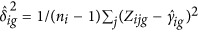 and 
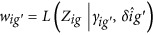


(3) The method adjusts the data for batch effects


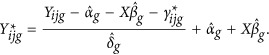


using estimated batch effects 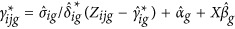


Lastly, we evaluated mixture approaches by combining PCA with empirical Bayes method: first, we removed unwanted variance from irrelevant factors and then tried to identify remaining noise and systematic bias using non-parametric Bayes method. Different classification algorithms were subsequently used on the different combinations.

### Feature reduction and filtering

Several filtering scores were compared in order to select the best-performing method for each classification algorithm. Average classification accuracy and its s.d. were obtained from 200 random, independent resamplings. For each resampling step, the data set was split 80/20 into training and test sets, respectively, and the features with the highest scores on the training set were selected. The model was then built on the training set and performance was evaluated on the test set. The following filtering scores have been compared and the best-performing methods for support vector machine and RF classification algorithms were carried forward for the purposes of analysis:

- *T-statistic:* The absolute value of Welsh's *t*-test statistic value. In case of a two-class classification problem, *t*-statistic is equivalent to the difference between two means for each class adjusted for s.d. for both classes.
- *Mean difference:* The absolute value of the difference between average values in each class.
- *P*-value for Welsh's *t*-test with degrees of freedom.
- *Correlation:* Absolute value of Pearson correlation between a variable and the predicting factor.
- The package ‘gene-filter' for R, available from Bioconductor, has been used to apply the above filtering criteria.^22^
- *P*-value *Limma:* The *P*-value calculated for the single-variable regression model using Limma. Bioconductor limma R package has been used for computing this score.^23^
- *RankProd:* RankProd modifies and extends the rank product method proposed by Breitling *et al.* ^24^ to integrate multiple studies from different laboratories and/or platforms. In case of pairwise ratios, for each gene it calculates feature rank for each comparison and returns—in this case, a log ratio of methylation levels in twins pair. Rank product is a geometric mean of ranks under different comparisons. The method has been implemented using the 'RankProd' R package.^25^

### Statistical learning

Two machine-learning classification approaches differing in several key aspects, namely linear SVMs and RF, were used to evaluate the effectiveness of the methods chosen to remove batch and population stratification effects and to classify affected from non-affected siblings based on methylation variation.^26, 27^ SVM and RF have parameters that need to be tuned. The linear SVM was implemented using a soft margin parameter C. RF has a number of variables randomly sampled as candidates at each split and used as parameter. The tuning and selection of parameters is required for both classifiers.

Linear SVM is a powerful method with an efficient computation time for a large number of variables and a small number of samples. As many other *ℓ*_2_ norm classification methods, SVM is sensitive to noise coming from all variables used. Selecting relevant features and decreasing the number of features aids noise reduction. The linear SVM model used has a soft margin parameter C, which is responsible for misclassification penalties. Higher values of parameter C forces the SVM to a higher penalty of misclassification on a training set that could lead to a higher generalization error if the final performance is only marginally above chance. Lower values of C could lead to lower generalization error. A soft margin parameter of *C*=0.05 was chosen following cross-validation.

RF has an embedded control for sensitivity to noise, but it is computationally inefficient for a large number of variables. Feature selection aids computation efficiency with additional noise reduction achieved by removal of irrelevant features, and therefore it is particularly important for large data sets using RF. RF has a tuning parameter, which is effectively the ratio of the number of variables randomly sampled as candidates at each split over the total number of variables. Control over the number of variables at each split aims to avoid correlation between trees in a forest. Prediction of a single tree is highly sensitive to noise in a training set, whereas average prediction across many trees is less sensitive if trees are not correlated. Different ratios for the RF model (with top 100 features selected by limma *P*-value) were evaluated and the ratio=0.1 was chosen as it returned higher average accuracy over 200 resampling.

Average classification accuracy as a measure of classification performance and its s.d. was obtained from 200 random independent resamples. At each resampling step, the data set was split into a training and a test set using a 80/20 ratio; features with the highest scores on the training set were selected. A model was built on the training set and performance evaluated on the independent test set. Consolidated data set were compared against the following unadjusted data sets:

- *Australian*, *the Netherlands* and *UK*—classification accuracy of the three regions was evaluated independently.
- *RAW data*—raw data from each region were merged without applying non-i.i.d. consolidation methods.
- *NormMean*—the mean normalized raw data before merging (for each data set, the mean normalization subtracts average values for each feature).
- *NormScale*—the mean and s.d. normalized raw data before merging (for each data set, the mean and s.d. normalization subtract average values for each feature and divide by s.d.).

The mean normalization and scaling aims to remove inequality between data sets in cases where for each epigenetic marker relative values between samples remain similar across different data sources, whereas the absolute level could vary together with variance. The above methods provide a baseline to evaluate the increase in performance following correction methods for violation of non-i.i.d. assumptions.

### Network analysis

In order to gain further biological insight into the most predictive features, MetaCore (https://portal.genego.com/) was used to explore the intersection between the list of genes mapped to variants uncovered from this study and known pathway maps and networks. Features most likely to be selected following feature reduction methods and that yielded the highest classification accuracy were mapped to known genes using the UHN Microarray Centre's CpG Island instrument (https://www.pmgenomics.ca/cpg). MetaCore scores and prioritizes networks and pathway based on the relevance of the genes uploaded. The gene list returned by the analysis presented may point at genes that show potential interaction, differential expression and may be involved in the pathology. The gene list could also potentially point at possible targets for therapeutic drug discovery. MetaCore evaluates the magnitude of the intersection between the reference gene list and the set of genes corresponding to a network module and returns different metrics including *P*-values and *G-scores*. *P*-values are calculated based on hypergeometric distributions, and these are used to establish whether saturation with the genes of interest is higher than random. When exploring signaling cascades, it is possible to evaluate whether a network contains any fragments of well-understood (canonical) signaling pathways. The *G-score* is another metric used by the software, which effectively modifies the *Z*-*score* based on the number of the linear canonical pathway fragments contained within the network. A high *G-score* therefore points at a network highly saturated with reference genes and containing several canonical pathways. In this study we have explored the top-ranking networks by *P*-value and by *G-score*.

### Code availability

All code including R scripts used to generate this analysis are available from the corresponding author on request or can be downloaded online (http://www.adamlab.org/epigenetics-of-depression/).

## Results

**Figure 1:**
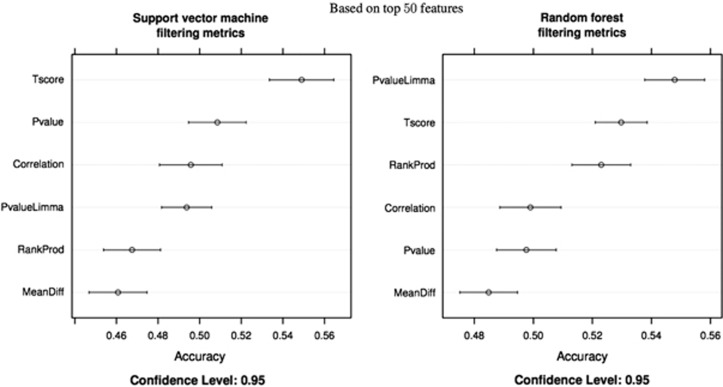
Linear support vector machines and random forest performances with features selected using different filtering scores. *t*-statistics is the feature reduction method that yielded higher classification accuracy for support vector machine (SVM), whereas Limma *P*-value showed better performance using random forest (RF). The results are based on top 50 features.

Data resulting from high-throughput technologies tend to be high-dimensional with many more variables than cases. Methods that can inform on the most relevant features and reduce the dimensionality of the data by identifying variants with larger effect size are used to offer a more favorable signal/noise ratio and to reduce model over-fitting. As each data set has unique structures and substructures, we first empirically evaluated different methods for feature selections for each of the two classification methods used. The results of our evaluation showed that the *t*-test statistics scoring metric was the filtering method that gave the best performance when using an SVM while single-variable linear model *P*-value yielded better-accuracy performance when using RF. Other scores such as correlation, *P*-value and mean difference showed comparatively worse performances (Figure 1). These two methods were carried forward for classification analyses.

**Figure 2:**
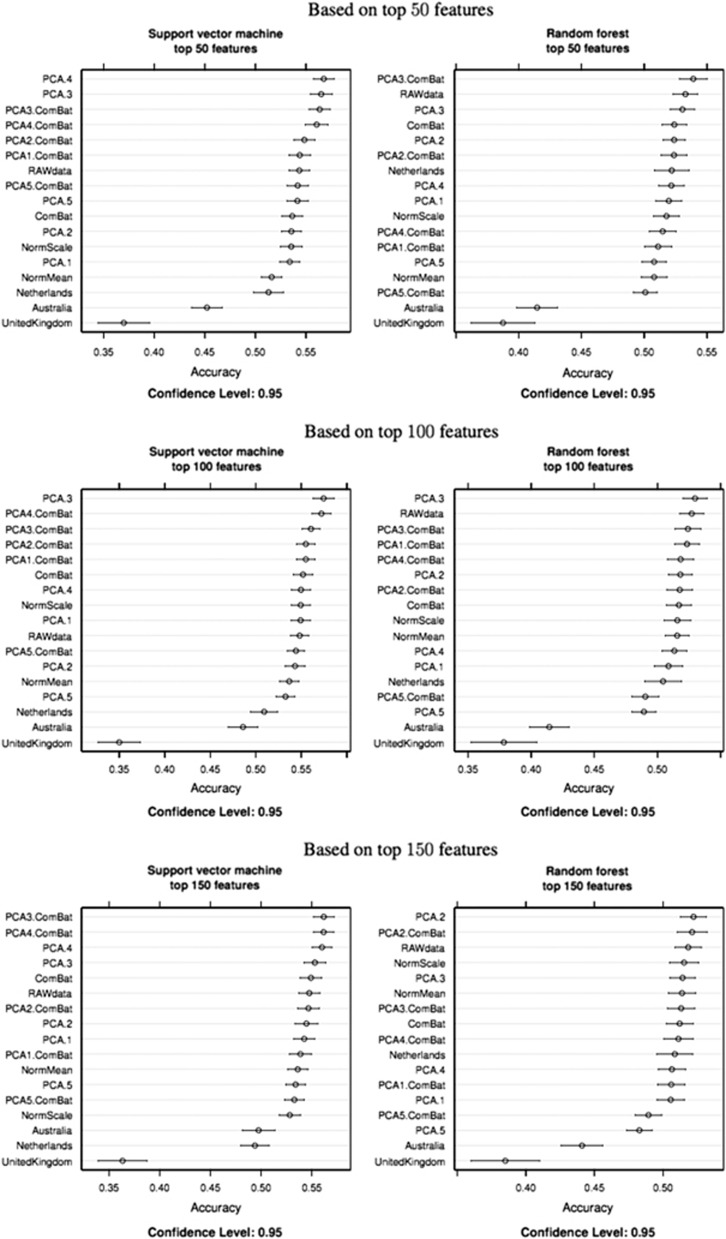
Summary of results for two classification methods (support vector machines and random forest) across different methods to control for non-independent-and-identically distributed (non-i.i.d.) data and different depths of feature selection. The mean accuracy percentage scores are reported on the *x* axis. It is not possible to classify cases and controls by analyzing each study individually, even after feature selection. The UK sample, the smallest, performs considerably worse compared with the other two, which show around chance levels. By leveraging the increased power offered by the combined samples and removing potential batch and noise variance it is possible to detect a weak but stable and significant signal. The highest classification accuracy (58%) is achieved with 100 features.

Analysis using SVM and RF in each of the three studies independently (UK, Australia and the Netherlands), with and without feature selection, returned overall poor classification accuracy. It was not possible to classify cases above chance level across any of the studies. The worse model performance was obtained in the UK study likely because of the smaller sample size compared with Dutch and Australian studies. The Dutch and Australian studies performed better in comparison with the UK study but were also underpowered and returned accuracy scores around chance levels (Figure 2). We then repeated the analyses using the three samples combined together. However, without correction to overcome issues of non-i.i.d. and removal of potential confounding effects, the combined sample produced a classifier that still did not perform better than chance, even when feature reduction was used (Figure 2). This is consistent with results reported by Oh *et al.* Feature reduction was an important step to improve the performance of the two algorithms but only after various correction methods applied to control for the non-i.i.d. nature of the data. With dimensionality reduction and correction methods, accuracy improved with the highest-accuracy performance achieved with a set of 100 features.

When evaluating different methods to control for confounding effects, *PCA.3* and mixture models using *PCA3.ComBat* showed a steady improvement of performance over raw data set for both linear SVM and RF models with different numbers of selected features. Methods with one more or one less number of removed principal components such as *PCA.4* and *PCA.2* with or without ComBat methods returned better accuracy for some classifiers, but were not steady across different numbers of selected features and different models using independent training and testing sets (Figure 2).

Mixture models using PCA.3 together with ComBat and PCA.4 together with ComBat were the best-performing methods when using a SVM, whereas mixture methods using PCA2, ComBat and PCA.2 returned the best performance using RF. The best overall classification was achieved using SVM and either removing the first three principle components or with a mixture approach using PCA3 and ComBat in conjunction with feature elimination. A classification of 58% was achieved from epigenetic variations in blood with 100 features selected. The results are significantly above chance level and remained above 57% even when the number of features was reduced to 50. The top-ranking features selected across the three different studies following resampling are summarized in Table 1. The signal that can be detected from blood clearly comes from a very small subset of the total number of features. However, this may be expected, given that the data was obtained from a peripheral tissue as opposed to a more disease-related one.

**Figure 3:**
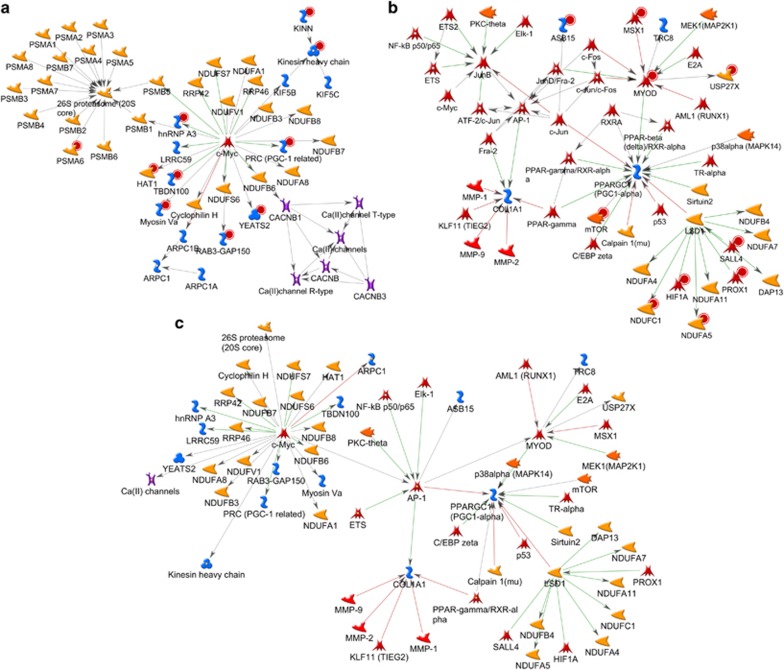
Top ranking gene networks. (**a**) The first score (by *P*-value) network from the study. Reference genes are marked with red circles. The network is centered on the c-Myc gene hub. C-myc is known as a proto-oncogene and is associated with cell proliferation and as a pro-apoptotic molecule. (**b**) The second scoring (by *P*-value) network from the study. The network includes 10 reference genes that are marked with red circles. The network is centered on the PPARG (human accelerated region 20 (HAR20)). Peroxisome proliferator-activated receptor-gamma (*PPAR-γ)* is a transcriptional coactivator involved in the regulation of energy metabolism and in mitochondrial biogenesis. Importantly, the gene has a role in the regulation of cAMP response element-binding protein (CREB). The transcription factor CREB has been implicated in signaling pathways relevant for pathogenesis and therapy of depression, which includes the c-Jun N-terminal kinases (JNKs). (**c**) Merging of the two top-ranking networks ranked by *P*-value reveal that the two central gene hubs (c-MYC and *PPAR-γ*) are linked by the activator protein (AP)-1-binding hub. The AP-1 is associated with dimeric transcription factors composed of Jun and Fos subunit. The Ap-1 target is particularly relevant for depression as it regulates gene expression in response to different stimuli including cytokines. Together with the nuclear factor (NF)-kappaB, AP-1 controls T-cell activation, followed by binding of foreign antigens to the T-cell receptor leading to cytokine secretion. AP-1 therefore has a key role in the initiation inflammatory response by activating immune cells through expression and secretion of chemokines and cytokines. Inflammation is an event that has been associated with increased risk of major depressive episodes. Higher levels of peripheral inflammatory markers taken from blood samples have been found in depressed patients.

A network analysis using MetaCore was peformed in order to gain additional insight into the potential relationship between the feature-set uncovered from the above analysis. The top-ranking genes that could be mapped to probes on the 8.1-K array with higher probability of being selected across the different resampling were uploaded as reference molecule to MetaCore's database. We first explored the top two ranking networks returned by *P*-value. The first ranking network (*P*<1.29 × 10^−21^, G-score=48.28) was centered on the *c-MYC* gene hub (Figure 3a). *C-MYC* is known as a proto-oncogene and is associated with cell proliferation and as a pro-apoptotic molecule. The second network by *P*-value (*P*<1.62 × 10^−21^, G-score=47.79) includes 10 reference genes and is centered on the *PPARGC1A* (human accelerated region 20 (*HAR20*)) gene hub (Figure 2b). The gene is a transcriptional coactivator involved in the regulation of energy metabolism and in mitochondrial biogenesis. Importantly, the gene has a role in the regulation of cAMP response element-binding protein. The transcription factor cAMP response element-binding protein has been implicated in signaling pathways relevant for pathogenesis and is associated with the c-Jun N-terminal kinases. The network points at potential mechanisms involved in inflammation, which have been extensively associated with MDD. The two networks were subsequently merged in order to gain further insight into their potential relationship and interactions (Figure 3c). Interestingly, the gene hubs are one interaction away and modulated through a pathway centered on the AP-1 hub (*C-JUN*). c-Jun, in combination with c-Fos, forms the AP-1 early-response transcription factor. Activation is dependent on double phosphorylation by the c-Jun N-terminal kinase pathway, which has an important role in initiating inflammatory cellular responses.^28^

**Figure 4:**
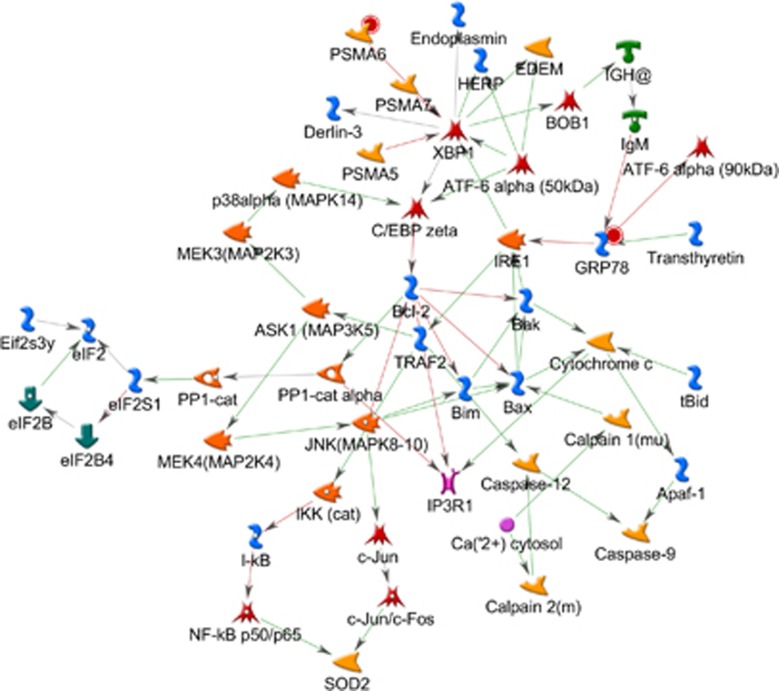
The highest-ranking network by G-score. The network reveals a number of potential interaction that may be relevant to the disease and have been previously associated with inflammation. The bottom of the network shows a cluster of genes including *c-Jun*, *Fos*, *AP-1* (together forming the APP complex), *JNK* (MAPK) and *NF-κβ*. On the upper part, the the network is clustered around the *XPB-1* gene hub, which is involved in modulation of the cellular response to endoplasmic reticulum (ER) stress brought about by stressful stimuli.

Lastly, we explored the top-ranking network by G-score (Figure 4). The network reveals a number of interesting genes including *c-Jun (AP-1)*, with interactions between c-Jun N-terminal kinase (MAPK) and the X-box-binding protein 1 (*XPB-1*) gene. The *XBP1* gene encodes a key transcription factor in the unfolded protein stress response and has been implicated in the pathophysiology of MDD.^29^

## Discussion

Landscapes of DNA methylation can show great variation between different tissues, but accessibility to brain tissues in human studies is limited to post-mortem and surgical resection. Therefore, peripheral tissues, including blood, can be an important source of information for the identification of biomarkers and mechanisms associated with the pathology that are assumed to also be manifested in the brain. However, disease-associated epivariations in blood may be less pronounced, more sparsely distributed and complicated to detect because of noise variance compared to disease-affected brain tissues. In this study we combined three non-i.i.d. data sets using a data-driven approach to uncover a suitable method to control for both batch effect and noise variance in order to build a global classifier that could classify affected siblings in MZ pairs discordant for MDD from epivariation in peripheral blood.

Of the range of methods evaluated for non-i.i.d. data consolidation, PCA with the top three eigenvector methods and mixture approaches using PCA and ComBat showed steady positive results on our data sets. The resulting model could classify the disease state above chance levels in an independent testing set using differences in methylation levels. Out of several methods considered for classification, the linear support vector machine with the top 100 selected features returned the highest classification accuracy. The accuracy prediction above chance level can be considered important, given the complex molecular architecture underpinning the pathology. Accuracy measures were reported instead of receiver operating characteristic scores, even if these can often be higher. Indeed, high sensitivity and specificity can be achieved even in the absence of high accuracy. Being prudent in quantifying the ability to discriminate discordant twins for MDD from epigenetic markers in peripheral blood alone was preferred to reporting higher ROC scores. This is particularly important in the context of disease classification where it is likely that accumulation of epigenetic changes may explain only a small fraction of individual differences in the pathology. Enrichment analysis of the top-ranking features points at an epigenetic signature of MDD that can be detected in peripheral blood and may be used to inform candidate gene selection in future molecular studies of MDD.

The results of the analysis identified a number of key genes, which have previously been shown to be involved in the pathophysiology of MDD. These genes include *PPAR-γ (*peroxisome proliferator-activated receptor-gamma), *AP-1*, *XPB-1* and *NF-κβ*. One of the gene maps uncovered from this study focused around *PPAR-γ* as the center gene hub (Figure 3b). Merging the two top-ranking networks showed that *AP-1* appears to be a connection molecule mediating between the two gene hubs of the two networks; these two top-ranking networks by *P*-value appear to be one interaction away. It is possible that DNA methylation may modulate a number of regulatory processes affecting these networks. The precise mechanisms by which these epigenetic changes may affect the pathophysiology of depression or whether these can be used as potential biomarkers from peripheral fluids needs further exploration.

### *AP-1* and NF-κβ

The results from this study support previous human and animal studies on MDD that have uncovered genes centered around a stress-response cascade involving the activator protein 1 *(AP-1)* and nuclear factor kappa-light-chain-enhancer of activated B cells (*NF-κβ*).^30^ *AP-1* is a gene that codes for a transcription factor that regulates gene expression in response to cytokines as well as environmental stress and bacterial and viral infections. *AP-1* downregulation has been implicated as part of the mechanism by which administration of IFN-alpha therapy induces depression symptoms.^31^ *NF-κβ* is a transcription regulator that has a role in peripheral inflammation with both pro- and anti-inflammatory effects.^32, 33^

### *PPAR-γ*

One of the loci uncovered by the network pathway analysis is the *PPAR-γ* gene that codes for the glitazone receptor (NR1C3). Activation of the *PPAR-γ* system in the central nervous system is thought to decrease parainflammation, endoplasmic reticulum (ER) stress, formation of reactive oxygen species and glutamate toxicity while increasing neurogenesis and neuroplasticity.^34^ It has been shown that activation of *PPAR-γ* leads to an increased neurogenesis as well as antidepressant effects in rodent models.^35, 36^ While the mechanisms by which this leads to an antidepressant effect are unclear, it has been shown that exposure to stress is associated with decreased hippocampal neurogenesis. Induction of neurogenesis has been shown to be a mechanism of action of several antidepressant medications in animal models.^37, 38, 39^

Interestingly, several clinical cross-over and randomized control trials have evaluated the efficacy of the insulin-sensitizing *PPAR-γ* agonists thiazolidinediones (troglitazone, pioglitazone and rosiglitazone) for the treatment of patients with concomitant MDD and metabolic syndrome or diabetes, as adjunctive therapy in patients with moderate-to-severe MDD in the absence of other metabolic disorders and as monotherapy.^40, 41, 42, 43^ In addition to their action as insulin-sensitizing agents, these drugs also have anti-inflammatory, neuroprotective and anti-excitotoxic properties.^44^ Activation of PPAR-γ receptors by their natural (15d-PGJ2) or synthetic ligands has been shown to support neuronal glucose and glutamate metabolism following exposure to stress and to increased levels of neurotropic factors.^45, 46^ These processes are believed to be dysregulated in MDD, thus making PPAR-γ activation a drug target of interest.

### XPB-1

The top-ranking network by G-score revealed a network with the *XPB-1* as a principal hub (Figure 4). *XPB-1* is involved in the cellular response to ER stress brought about by stressful stimuli. ER stress occurs when demands on the ER to fold and process proteins are increased beyond its capacity, leading to the production of unfolded proteins.^47^ This initiates the ER-stress response, which promotes protein folding and secretion, as well as unfolded protein degradation. Failure of this response leads to calcium ATPase (Ca2+) release from the ER, leading to cell apoptosis. XPB-1 activates unfolded protein degradation as well as production of chaperones for protein secretion.^34^ A polymorphism leading to decreased *XPB-1* expression has also been implicated in bipolar disorder.^48^ Sodium valproate, an anticonvulsant with mood-stabilizing properties, affects the ER-stress response, reducing the likelihood of cell apoptosis. One mechanism of action for this is the activation of *ATF6*, the gene that activates *XPB-1* in the hippocampus and the cerebral cortex.^49, 50^

### Strengths and limitations

DNA methylation is known to exhibit tissue specificity, but human studies are limited to either post-mortem, surgical resection or often surrogate tissues, which include blood cells. The extent to which epivariations in surrogate tissues resemble those in central tissues of interest is unknown. Given the complexity of the pathology, it is likely that many epigenetic variants involved in a number of mechanisms underpinning MDD cannot be detected in blood. On the other hand, blood cells may show fewer epigenetic changes associated with factors associated with the disorder (such as in response to pharmacological treatment) but not involved in etiology. According to the peripheral inflammation hypothesis of depression, it is systemic inflammation that could potentially explain part of the molecular etiology of the disorder. Many of the inflammatory molecules implicated by this hypothesis are detectable in peripheral blood cells. The most predictive epigenetic markers uncovered by this study were mapped to genes and gene networks with mechanisms associated to inflammation, which is reasonable to expect from blood. It is, however, unclear whether peripheral inflammation is involved in the etiology of depression or is purely driven in response to it.

This study was able to control for the non-i.i.d. nature of the data, allowing the integration of three separate data sets, each from a different center and country, overcoming one of the limitations from a previous published study using the same three data sets. In isolation, the studies were too underpowered to allow classification of affected and non-affected siblings, but leveraging the increased power of the larger sample allowed detection of a clear signal. Independent testing and training and careful optimization of parameters to avoid model over-fitting increased the probability of true-positive findings. Although classification was possible above chance levels from peripheral blood, classification accuracy remains generally low. On the other hand it is likely that only a small portion of the phenotypic variance is explained by epigenetic variations. However, this approach allowed us to explore the etiology of MDD beyond gene + environment and gene × environment etiological paradigms.

This method of modeling allows the identification of epigenetic loci that account for a portion of the variance in phenotype between MZ twin pairs discordant for depression. Whereas these loci can be identified, this methodology does not give any information as to their role. The construction of networks of epigenetic loci identified from our analysis revealed which epigenes are linked, suggesting pathways and relationships between the proteins these genes code for. The genes implicated by the model can then be corroborated with the literature, shedding light on their role in MDD pathophysiology, although this is inevitably subjected to bias. Furthermore, the majority of the literature does not look at the role of specific genes from the epigenetic perspective; therefore, there is potential for further research involving genes both implicated in the pathophysiology of depression, such as the peripheral inflammation hypothesis, and the epigenetics of depression.

Many of the genes identified in the networks of this analysis are yet to be included in any literature regarding the pathophysiology of depression. *C-Myc* is a good example of this (Figure 3a). It is an oncogene implicated in the pathophysiology of a number of cancers, but it has not yet been researched extensively in the field of psychiatry.

This study employs a cross-sectional design, meaning that it is not possible to infer causality. Accessibility of peripheral blood samples from the same MZ twin pairs over time could allow to identify changes in DNA methylation in relation to time of onset of depression and potentially identify a causal link.

## Figures and Tables

**Table 1:** The table reports the array probes that have higher probability of being selected following cross-validation

*UHNID*	*Genome location*	*Within strand*	*Gene symbol*	*Upstream strand*	*Gene symbol*	*Downstream strand*	*Distance*	*Gene symbol*
UHNhscpg0013724	chrX:49643159-49643620	−	*hCG_1993022*	+	*PAGE4*	+	849	*USP27X*
UHNhscpg0014299	chr1:220700559-220701079			−	*RAB3GAP2*	+	488	*KIAA1477*
UHNhscpg0013480	chr15:42564937-42565851	−	*TMEM87A*	−	*VPS39*	+	4	*GANC*
UHNhscpg0000046	chr10:44405717-44405979			−	*HNRPA3*	+	28471	*AX747950*
UHNhscpg0004659	chr18:57566852-57567954	+	*PMAIP1*	−	*CCBE1*	−	470609	*MC4R*
UHNhscpg0014315	chr13:108325128-108325573	−	*FAM155A*	−	*ARGLU1*	−	114136	*BC043519*
UHNhscpg0014319	chr2:143972533-143972830	+	*ARHGAP15*	+	*KYNU*	+	220332	*ARHGAP15*
UHNhscpg0003893	chr19:41073929-41074621	+	*SPTBN4*	+	*SPTBN4*	+	8135	*SHKBP1*
UHNhscpg0000830	chr20:11309478-11309784			−	*BC043370*	+	561692	*BTBD3*
UHNhscpg0005193	chr15:75871291-75871420	−	*PTPN9*	+	*LENG6*	−	19005	*SNUPN*
UHNhscpg0014339	chr7:63774121-63774927	+	*AK301806*	+	*ZNF679*	+	38441	*AK123141*
UHNhscpg0014256	chr9:109514015-109514290			+	*AK093363*	+	111087	*ZNF462*
UHNhscpg0000120	chr17:8029524-8030292			−	*HES7*	−	13496	*hPer*
UHNhscpg0014546	chr3:121553705-121554108	−	*IQCB1*	−	*GOLGB1*	+	59062	*SLC15A2*
UHNhscpg0013060	chr6:133561543-133562818	+	*EYA4*	+	*LOC285735*	−	193397	*AK093513*
UHNhscpg0002422	chr11:131557833-131558280	+	*NTM*	−	*AK128059*	+	221219	*NTM*
UHNhscpg0013741	chr13:73355948-73356559	−	*DIS3*	+	*C13orf34*	+	272554	*KLF5*
UHNhscpg0014277	chr7:123197380-123198004	−	*NDUFA5*	−	*IQUB*	+	43916	*ASB15*
UHNhscpg0014284	chr15:52860388-52860692	−	*ARPP19*	−	*MYO5A*	−	12830	*DKFZp666F111*
UHNhscpg0017254	chr4:56221111-56221294	+	*SRD5A3*	−	*KDR*	−	8845	*BC047403*
UHNhscpg0016077	chr19:57350030-57350493	−	*ZIM2*	−	*ZIM2*	+	1776	*MIMT1*
UHNhscpg0000829	chr5:121412422-121413979	−	*LOX*	−	*LOX*	+	51235	*ZNF474*
UHNhscpg0014173	chr11:17694766-17694956			+	*OTOG*	+	46153	*MYOD1*
UHNhscpg0004403	chr10:103880275-103880427			−	*LDB1*	+	12359	*PPRC1*
UHNhscpg0013749	chr3:183542146-183542612	−	*MAP6D1*	+	*YEATS2*	−	4561	*PARL*
UHNhscpg0014254	chr2:166103020-166103587	+	*SCN2A*	−	*SCN3A*	+	46753	*SCN2A*
UHNhscpg0006419	chr1:24969946-24970475	+	*SRRM1*	+	*C1orf130*	+	4776	*SRRM1*
UHNhscpg0003218	chr14:35761163-35761287	+	*KIAA0391*	+	*KIAA0391*	+	286	*PSMA6*
UHNhscpg0007062	chr6:35265208-35266730	+	*DEF6*	+	*ZNF76*	−	41098	*BC016143*
UHNhscpg0003949	chr10:14287820-14288200	−	*FRMD4A*	−	*FRMD4A*	−	79973	*KIAA1294*
UHNhscpg0018256	chr2:172778466-172778887			−	*SLC25A12*	+	47	*HAT1*
UHNhscpg0013413	chr14:36741419-36741890			+	*BRMS1L*	−	25873	*MBIP*
UHNhscpg0015148	chr3:61236979-61237436	−	*FHIT*	−	*NPCR*	+	309806	*PTPRG*
UHNhscpg0013509	chr4:140223250-140223792	+	*NARG1*	−	*NDUFC1*	+	151168	*RAB33B*
UHNhscpg0005075	chr22:43010321-43011102	−	*POLDIP3*	−	*KIAA1649*	−	3711	*CYB5R3*
UHNhscpg0014262	chr21:43683519-43684423	+	*ABCG1*	+	*UMODL1*	+	21566	
UHNhscpg0014278	chr11:64216985-64217570			+	*BC038767*	+	105527	*hOAT4*
UHNhscpg0014576	chr20:50719871-50720391	−	*ZFP64*	−	*SALL4*	−	47428	*ZFP64*
UHNhscpg0002409	chrX:128912982-128913773			+	*XPNPEP2*	+	118	*SASH3*
UHNhscpg0015607	chr9:33473226-33474250	−	*SUGT1P*	−	*NOL6*	+	74151	*BC009440*
UHNhscpg0016477	chr1:11322016-11323073	−	*MTOR*	+	*ANGPTL7*	+	10181	*UBIAD1*
UHNhscpg0011113	chr4:4859613-4859967			+	*AK056081*	+	1424	*MSX1*
UHNhscpg0015879	chr12:48299099-48299618			−	*NR1I1*	+	57711	*TMEM106C*
UHNhscpg0015606	chr12:57957633-57958195	+	*KIF5A*	−	*DCTN2*	+	20493	*BC033961*
UHNhscpg0014519	chr1:214159322-214159791			−	AK092251	+	1494	*PROX1*
UHNhscpg0016016	chr14:62228816-62229592	+	*SNAPC1*	+	*HIF1A*	+	224210	*syt14r*
UHNhscpg0002174	chr12:45444911-45445553			−	*DBX2*	−	10849	*FKSG42*
UHNhscpg0005059	chr21:32929750-32930539	−	*TIAM1*	−	*TIAM1*	+	1018	*BC014150*
UHNhscpg0001248	chr10:97890139-97890702	+	*ZNF518A*	−	*BC028619*	−	60760	*BLNK*
UHNhscpg0000005	chr10:1094361-1094422	−	*IDI1*	−	*IDI1*	+	574	*BC046483*

Column one shows array probe ID. Column two shows genome location including chromosome number and distance. Columns 3, 5 and 7 show the directionality of within, upstream and downstream strand. Columns 4, 6 and 9 show the gene symbols mapped to the array probes.
